# Distribution of hepatitis B virus genotypes in the general population of Myanmar via nationwide study

**DOI:** 10.1186/s12879-020-05269-z

**Published:** 2020-07-29

**Authors:** Yi Yi Kyaw, Aye Aye Lwin, Khin Saw Aye, Hlaing Myat Thu, Moh Moh Htun, Hnin Ohmar Soe, Kay Thi Aye, Kyaw Zin Thant, Hyeon Jeong Hwang, JaeHun Cheong

**Affiliations:** 1 Advanced Molecular Research Centre, Department of Medical Research, Republic of Union of Myanmar, Yangon, Myanmar; 2 Department of Medical Research, Republic of Union of Myanmar, Busan, 609-735 South Korea; 3 Department of Molecular Biology, Pusan National University, Yangon, Republic of Korea

**Keywords:** Hepatitis B virus, Genotype, Sub-genotype, Myanmar

## Abstract

**Background:**

Hepatitis B virus (HBV) infections are a severe health concern worldwide. HBV is a DNA virus with a rapid rate of mutation. Based on heterogeneity of the nucleotide sequence, the HBV strains are divided into nine genotypes, each with a characteristic geographical distribution. Identifying and tracking alterations of HBV genotypes is important in epidemiological and transmission studies, and contributes to predicting the risk for development of severe liver disease and response to antiviral treatment. The present study was undertaken to detect HBV genotypes and sub-genotypes in the general population of different states and regions in Myanmar.

**Methods:**

In 2015, a total of 5547 adults of the general population, residing in seven states, seven regions and the Nay Pyi Taw Union Territory, were screened for Hepatitis B Surface antigen (HBsAg) by the immunochromatographic test (ICT). Of the 353 HBsAg positive samples, the HBVDNA was identified using polymerase chain reactions (PCR) targeting the DNA sequences encoding the Pre-S region. A total of 153 PCR positive samples were subsequently subjected to genotyping by partial genome sequencing in both directions. The resulting sequences were then edited, aligned, and compared with reference sequences using the National Centre for Biotechnology Information (NCBI) web-based genotyping tool.

**Results:**

Three HBV genotypes (HBV genotype B, genotype C and genotype D) were detected in Myanmar, of which genotype HBV genotype C (66.7%) was the most prevalent, followed by HBV genotype D (32%) and HBV genotype B (1.3%). Sub-genotyping revealed a total of 7 variants within the B, C and D genotypes: 2 (B4 and B5) in HBV genotype B, 3 (C1, C5 and C7) in HBV genotype C, and 2 (D3 and D6) in HBV genotype D.

**Conclusion:**

HBV genotype C, sub-genotype C1 was predominantly distributed in all states and regions of Myanmar. This study is the first report on the nationwide distribution of HBV genotypes and sub-genotypes in Myanmar. We believe our findings will enable huge support for the hepatitis disease surveillance program, since HBV infection is one of the National Priority Diseases in Myanmar.

## Background

The Hepatitis B virus (HBV) belongs to the genus *Orthohepadnavirus* of the *Hepadnaviridae* family and its liver infection can cause both acute and chronic diseases. Globally, an estimated 257 million people are living with hepatitis B virus (HBV) infection. In 2015, hepatitis B infection resulted in 887,000 deaths, mostly from complications that include cirrhosis and hepatocellular carcinoma [1]. In the same year, the World Health Organization (WHO) estimated that the global prevalence of HBV infections in the general population was 3.5%. The proportion of persons living with chronic HBV infection remains high among those born before availability of the hepatitis B vaccine. Prevalence was the highest in the African (6.1%) and Western Pacific regions (6.2%), followed by Asia (2%) [2]. Myanmar has a moderate to high endemicity of hepatitis B infection. According to the nationwide seroprevalence survey in 2015, 6.5% of the general population was infected with viral hepatitis B. The prevalence varied with geographic locations, with highest prevalence in the Yangon Region (10%) and lowest in Kayah State (4.2%) [3].

HBV is a circular DNA molecule of approximately 3.2 k base pairs; it is a partially double-stranded DNA that replicates through an RNA intermediate anti-genome sequence, using its own encoded reverse transcriptase (RT). Since HBV-RT is lacking in a proof-reading function, there are occurrences of error frequencies, and these error-prone conditions are similar to those encountered in retroviruses and other RNA viruses [4]. Persistent and long-term infections, and different selected pressures on viruses, has resulted in the emergence of HBV variants. Some of the variants are able to evade diagnostics as well as prophylactic and therapeutic measures. The HBV genome encodes viral proteins through four open and partially overlapping reading frames: surface (preS/S), core (preC/C), polymerase (P), and X genes. These genes encode for specific proteins: preC/C, for the e antigen (HBeAg) and core protein (HBcAg); P gene, for polymerase (reverse transcriptase); S gene, for surface proteins (there are three forms of HBsAg: small (S), middle (M) and large (L)); and X gene, for a transcriptional transactivator protein [5, 6].

Based on the genome sequence, HBV is grouped into numerous genotypes, of which 9 genotypes are well-defined. Some HBV genotypes are further classified as sub-genotypes. The HBV sequence is characterized by having more than 8% nucleotide differences for genotypes, and more than 4–8% nucleotide differences for sub-genotypes. To date, over 30 related sub-genotypes belonging to HBV genotypes have been determined [7, 8].

An earlier classification system divided the HBsAg into four major serological subtypes, viz., *adw, adr, ayw* and *ayr*, which, in turn, are correlated to HBV genotypes. In general, HBV genotypes of A, B, F, G or H have the HBsAg subtype *adw*, whereas HBV genotype C have *adr*, and HBV genotype D and HBV genotype E have *ayw* [9, 10]. Genotypes A and D are globally distributed, whereas genotypes B and C are predominantly found in east and southeast Asia, and genotype E prevails in West Africa. The most divergent genotype F is found exclusively among the indigenous people of central and south America. Genotype G, found in the USA and France, exhibits a unique molecular structure [8].

Myanmar is one of the most ethnically diverse countries, bordered by Bangladesh and India on the western border, China, Laos and Thailand on the eastern border, Thailand on the southern border, and China on the northern border. The major genotype of HBV in China and Thailand is genotype C, while the genotype D is most prevalent in India [4, 11–14]. There are limited studies in Myanmar on HBV serotypes and genotypes. Previous studies on distribution of HBV serotypes and genotypes in Myanmar were mainly carried out on specific populations. A study in 2012 reported the distribution of HBsAg subtypes among the HBV carriers in Yangon as *adr* (93.2%), *adw* (4.85%) and *ayw* (1.94%) [15]. A hospital-based study showed HBV genotype C as the prevalent HBV genotype in chronic liver disease, followed by HBV genotype A, as well as mixed genotypes and unknown genotypes [16]. Sa-Nguanmoo et al. (2010) also reported the occurrence of HBV genotype C (97.5%), HBV genotype B and HBV genotype D (1.25% each) among Myanmar migrant workers in Thailand [17]. Recently, Latt et al. reported on whole genome sequences of 15 isolates from Myanmar HBV carrier patients, revealing that all were genotype C with sub-genotype C1 [18].

Genotypes and certain sub-genotypes have distinct geographical distribution, and are important in both clinical manifestation of infection and response to antiviral therapy. Moreover, the HBV genotype/sub-genotype and the inherent genetic variability are also useful in epidemiological and surveillance studies, tracing human migrations, predicting the risk of developing severe liver diseases, and responses to antiviral therapy [19]. However, there are no large-scale studies on the geographical distribution of HBV genotypes in Myanmar, and this study is, therefore, the first report on the nationwide distribution of hepatitis B genotypes and sub-genotypes.

## Methods

### Study site and study population

From May to October 2015, a cross-sectional survey was conducted in 18 townships of all states and regions of Myanmar. The 18 townships were selected from 7 states (Kachin, Kayah, Kayin, Chin, Mon, Shan and Rakhine), 7 regions (Bago, Sagaing, Magway, Ayeyarwady, Tanintharyi, Yangon and Mandalay), and the Nay Pyi Taw Union Territory. A total of 5547subjects, aged between 15 to 80 years and belonging to both genders, participated in the survey.

### Sampling procedure and recruitment

To achieve a national representative sample, the two-stage cluster sampling method was used. Selection of the primary sampling units (PSUs) was performed by randomly selecting one township, which was considered to have an average level of viral hepatitis B when considering all states and regions of Myanmar. Selection of secondary sampling unit (SSUs) was achieved by selecting 10 wards and villages from each selected PSU township, based on the probability to population size. Systemic random sampling was then used to select 30 households from each selected SSU (ward/village). The sampling frame for the current study included the list of households available to the Basic Health staff. One eligible participant in the selected household, aged between 15 to 80 years, was recruited by random sampling. Informed consent of the participant was obtained by the in-field investigators who explained the purpose and procedure of the study, and it was signed on-site before blood samples were drawn. Hepatitis B virus screening was carried out with the rapid assay SD Bioline HBsAg WB (Cat. No 01FK10W, Standard Diagnostic, Inc., Korea), and results were shared with the participants individually, in a closed envelope. Counseling for consequences of HB infection, and treatment options and health education was imparted to all positive patients, and a second informed consent form was obtained for genotyping study. All positive patients from sampling sites in Myanmar were invited for a genotyping study without sampling bias [3].

### Sample collection for genotyping study

The field investigators explained the purpose and procedure of the study, and informed consent was obtained from each subject before 2 ml venous blood samples were drawn. Sera were separated and transported to the Department of Medical Research for further genotyping. A total of 353 HBsAg positive subjects, 147 males and 206 females with mean age 35.5 years (SD = 10.8), were included in this genotyping study. The number of samples in this study represents a 99.7% response rate of the total 354 HBV sero-positive patients confirmed from the nationwide study.

### Confirmation of HBsAg positive serum samples

Serum samples which tested positive for HBsAg by ICT (Immuno-chromatographic Test) were further confirmed with a commercially available HBsAg ELISA 3.0 immunoassay kit (Cat. No 01EK10, Standard Diagnostic Test Kit, SD, Korea). The tests were performed according to the manufacturer’s instruction.

### Viral DNA extraction

Serum samples confirmed HBsAg positive by ELISA were subjected to viral DNA extraction, which was achieved with the QIAampDNA Mini kit (Qiagen, Inc., Hilden, Germany), according to the manufacturer’s instructions.

### Amplification of the preS gene of HBV by PCR

The HBV preS gene was amplified with nested PCR, using PF-PR and NF-NR primer sets (PF 5′ TTG GAC TCA CAA GGT GGG AA 3′; PR 5′ GTC CAC CAC GAG TCT AGA CTCT 3′; NF 5′ TCA TTT TGT GGG TCA CCA TAT 3′; NR 5′ CTG TAA CAC GAG CAG GGG T 3′). The primers were located in the preS/S genomic regions to ensure a high sensitivity for amplification of all HBV genotypes. The amplification mixture contained 5 μl extracted HBVDNA, Tris HCL buffer, 2 mM magnesium chloride, 0.1 mM dNTPs, 2 units taq polymerase (Cosmo), and 0.25 μM each of the primers. The PCR thermal cycling profile was as follows: 5 min at 94 °C, followed by 30 cycles comprising 30 s at 94 °C, 30 s at 51 °C, and 45 s at 72 °C, and finally 10 min at 72 °C. Negative samples after the first round PCR were amplified in nested PCR using the second round primer set and a thermal profile similar to the first round, but repeated for 35 cycles instead of 30, with annealing at 54 °C. After confirming 578 bp PCR product by gel electrophoresis, the products were purified with the SV column PCR purification kit (GeneAll Biotech, Korea), according to the manufacturer’s instructions.

### Determination of HBV genotypes by direct sequencing of preS gene

The purified PCR products were subjected to sequencing by chain termination method, using a commercially available kit (Big DyeTerminator Cycle Sequencing Kit, Applied Biosystems). Briefly, 2 μl purified DNA was mixed with 1.85 μl 5x sequencing buffer, 0.25 μl Big dye terminator, 0.5 μl 0.125 μM primer (forward or reverse) and 5.4 μl water. The thermal profile used was: 35 cycles comprising 60 s at 96 °C, 5 s at 50 °C, and 3 min at 60 °C. The 3500XL Genetic Analyzer (Applied Biosystems) was used for the Sanger sequencing method [20–22].

### Determination of HBV genotypes and sub-genotypes

HBV genotypes were determined by comparing with preS/S gene sequences of the NCBI Web based HBV Genotyping Tool (http://www.ncbi.nlm.nih.gov/ projects/genotyping/ formpage.cgi) [23]. HBV DNA sequences were aligned with reference sequences using the CLUSTAL method (MedAlign, Lasergene, DNASTAR Inc., Madison, WI). Sequences were manually edited with the BioEdit Sequence Editor (version 7.2.5), and phylogenetic relationships were established by applying the neighbor-joining method [24]. To confirm reliability of the pairwise comparison and phylogenetic analysis, bootstrap resampling and reconstruction were carried out 1000 times. For determination of the sub-genotype, study sequences were aligned with published sequences representing all known HBV sub-genotypes. Multiple sequence alignment was performed using the built-in ClustalW integrated in MEGA X software [25]. Phylogenetic analysis of HBV sub-genotypes was carried out using the MEGA X software. Genetic distances were calculated using the Kimura two-parameter model, and phylogenetic trees were constructed by applying the Maximum Likelihood method. The nucleotide sequences obtained in this study have been deposited at the NCBI GenBank database (Accession numbers: MH816993–995 and MH925817–925683).

### Statistical analysis

All statistical analyses were performed using the Statistical Program for Social Science Software (SPSS) 23.0 for Windows (SPSS Inc., Chigago IL., USA). Comparison between categorical variables was tested by the Chi-square test. Analysis of variance (ANOVA) was also performed to analyze the relationship of HBV genotype and HBV infection phase with age of the patients. A *P*-value (two tailed) of less than 0.05 is considered to be statistically significant. In this genotyping study, distribution of the HBV genotypes was compared among 5 geographical areas, such as central, east, north, south and western regions of Myanmar. Mandalay, Magway, Nay Pyi Taw, Bago, Ayeyarwady and Yangon regions were collectively described as the central area, Shan and Kayah states as the eastern area, Kachin state and Sagaing regions as the northern area, Mon and Kayin states and Tanintharyi region as the southern area, and Rakhine and Chin states as the western area. HBV sub-genotype results were subsequently analyzed collectively.

## Results

### Distribution of HBV genotypes among the study population

**Fig. 1:**
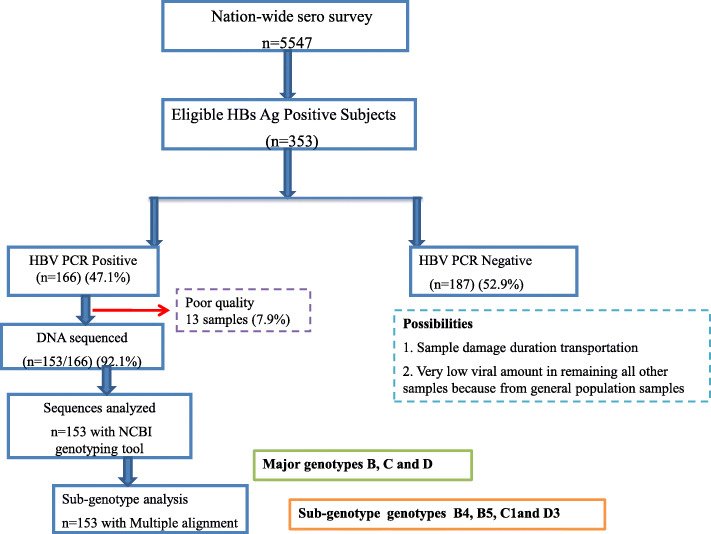
Flow Chart Diagram of HBV genotyping study

All 353 HBsAg positive serum samples also tested positive by HBV ELISA. Of the confirmed 353 HBsAg positive samples, 153 (43.3%) were PCR positive (69 males and 84 females, mean age 34.0 ± 11.54 years), and were further included for genotype analysis (Fig. 1). Three major genotypes (C, D and B) were found in this study population. The HBV genotype C (*n* = 102; 66.7%) was found to be the predominant circulating genotype (*p* = 0.002), followed by genotypes D (*n* = 49; 32%) and B (*n* = 2, 1.3%) (Table 1).

**Table 1:** Characteristics of subjects and Genotype and sub-genotypes distribution

Characteristics	Genotype B	Genotype C	Genotype D	Significance
Number of subjects (%) *n* = 153 (100%)	2 (1.3%)	**102** (66.7%)	**49** (32%)	*P =* 0.002
Age (Mean ± SD) 34.0 ± 11.4 Yr	41.5 ± 9.12	33.84 ± 11.72	33.88 ± 10.66	NS^b^ *P* = 0.065
Gender (M/ F) (69/84)	2/0	45/57	22/27	NS ^a^ *P* = 0.290
Sub-genotypes	2 B4,B5	3 C1,C5, C7	2 D3, D6	

^a^ Pearson Chi Square Test, ^b^Oneway analysis of variance, NS for not significant

### Distribution of HBV genotypes in the five geographical areas

**Fig. 2:**
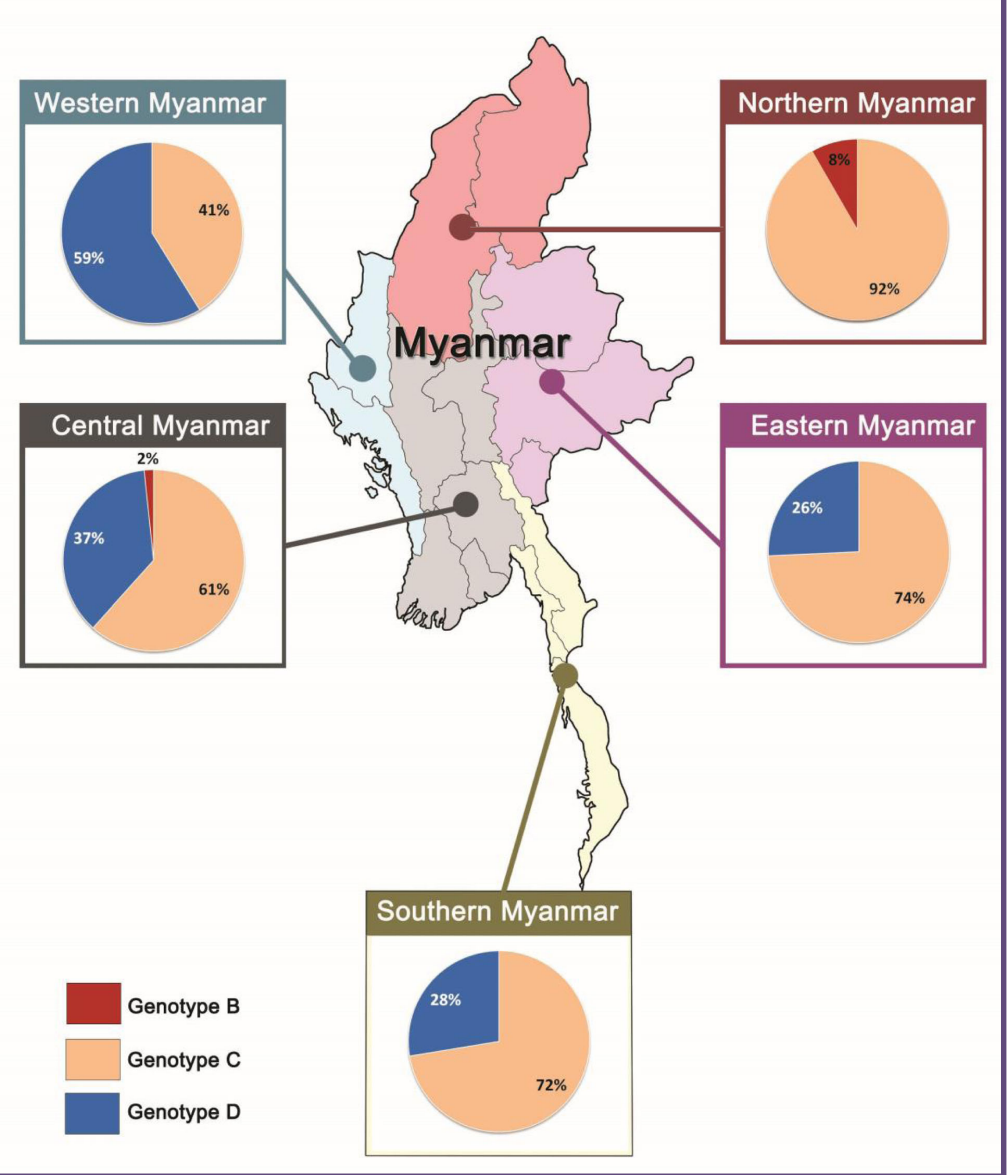
HBV genotype distribution in five geographical regions of Myanmar

The HBV genotypes were found differently distributed in the different regions of Myanmar (Table 2, Fig. 2). HBV genotype C was predominant in all areas, ranging from 61.7 to 91.7%, except in the western area (41.2%). Genotype B was found in two areas (north and central) with an occurrence of only 1.3% of HBV isolates. Genotype D was the major genotype (59%) in the western region of Myanmar, which borders with India and Bangladesh. HBV genotype C was predominant in the 35 subjects examined from the eastern area, and was identified in 26 (74.3%) subjects. Differential genotype distributions were observed in the western and eastern regions of Myanmar.

**Table 2:** Area- wise distribution of HBV genotypes in Myanmar

HBV Genotypes	Total Subjects	Area				
		Southern Area (Mon, Tanintharyi, Kayin states)	Western Area (Chin& Rakhine states)	Eastern Area (Shan& Kaya States)	Northern Area (Kachin states& Sagaing region)	Central Area (Mandalay, Magway, Nay PyiTaw,Ayeyarwaddi, Bago, Yangon)
		(*n* = 29), n%	(*n* = 17), n%	(*n* = 35), n%	(*n* = 12), n%	(*n* = 60), n%
C	102	21 (72.4%)	7 (41.2%)	26 (74.3%)	11 (91.7%)	37 (61.7%)
D	49	8 (27.6%)	10 (58.8%)	9 (25.7%)	0	22 (36.7%)
B	2	0	0	0	1 (8.3%)	1 (1.6%)
	**153**		NS

### HBV sub-genotypes in Myanmar

Among the 102 genotype C identified, the distribution of sub-genotypes was found to be HBV sub-genotype C1 (90.2%), followed by HBV sub-genotype C5 (5.9%) and HBV sub-genotype C7 (3.9%). In the total 49 HBV genotype D samples, majority were clustered into the HBV sub-genotype D3 (45, 91.8%), with the remaining identified as HBV sub-genotype D6 (8.2%). Only two HBV isolates were genotype B in our study population, belonging to sub-genotypes B4 and B5 (Table 2, Fig. 2).

**Fig. 3:**
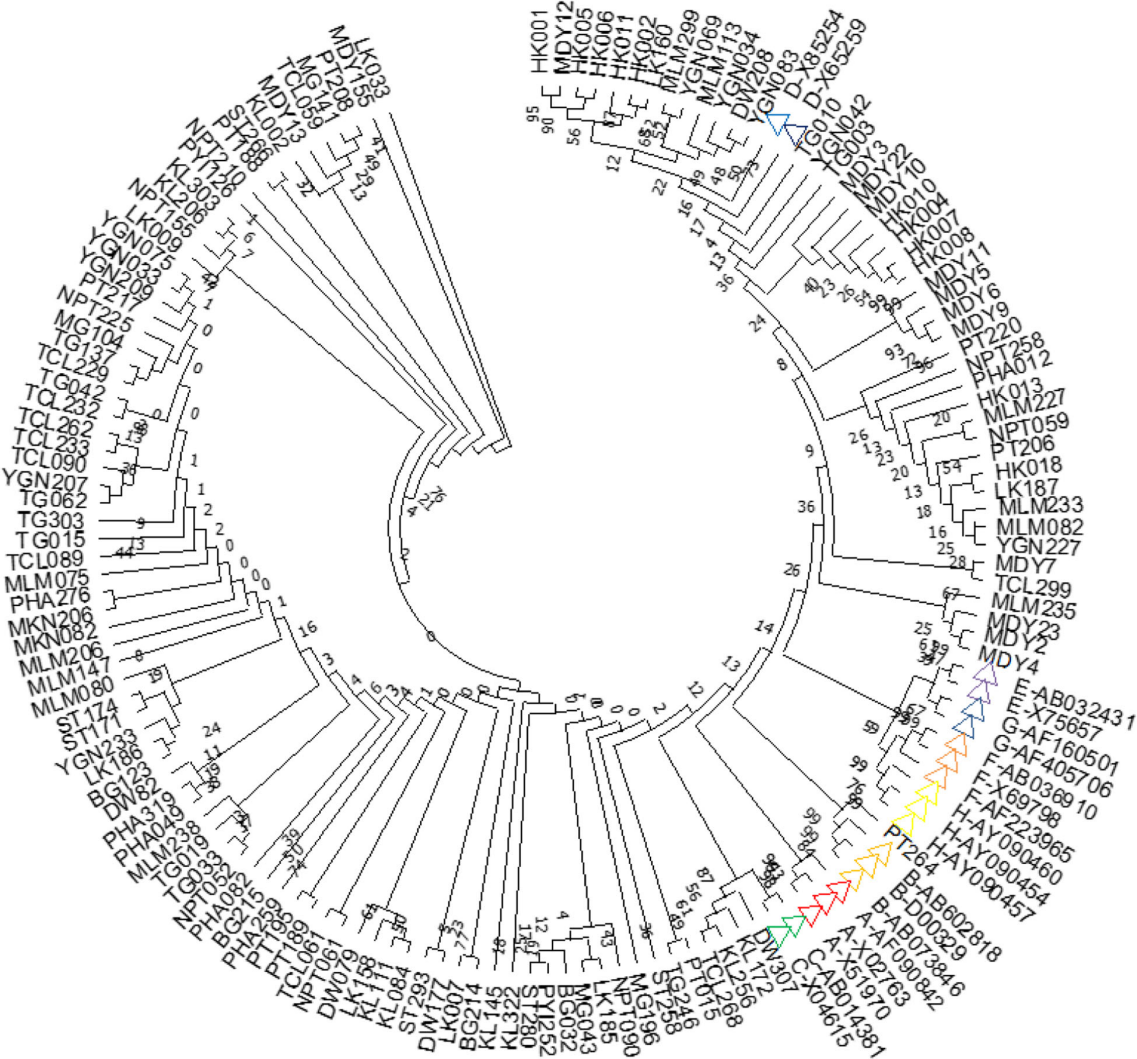
Cladogram of 153 HBV sequences with NCBI major genotype reference sequences. The phylogenetic tree was constructed using 578 bp nucleotide sequences (2860–222) PreS1/ PreS2 region of the reference genome of hepatitis B genotype representing the standard genotypes throughout the world. Phylogenetic analysis by neighbour-joining method with bootstrap test of 1000 replicates and maximum composite likelihood model was applied. Color triangles show the different reference major genotypes from the NCBI GenBank

Genotyping of the 153 HBV isolates (Accession numbers: MH816993–995 and MH925817–925683) was determined by constructing a cladogram (Fig. 3). The test sequences were grouped with reference sequences (Additional file 1) according to their genotypes and sub-genotypes. The genotypes of these sequences were also determined by the NCBI genotyping tool, which gave complete fidelity findings with the phylogenetic results.

**Fig. 4:**
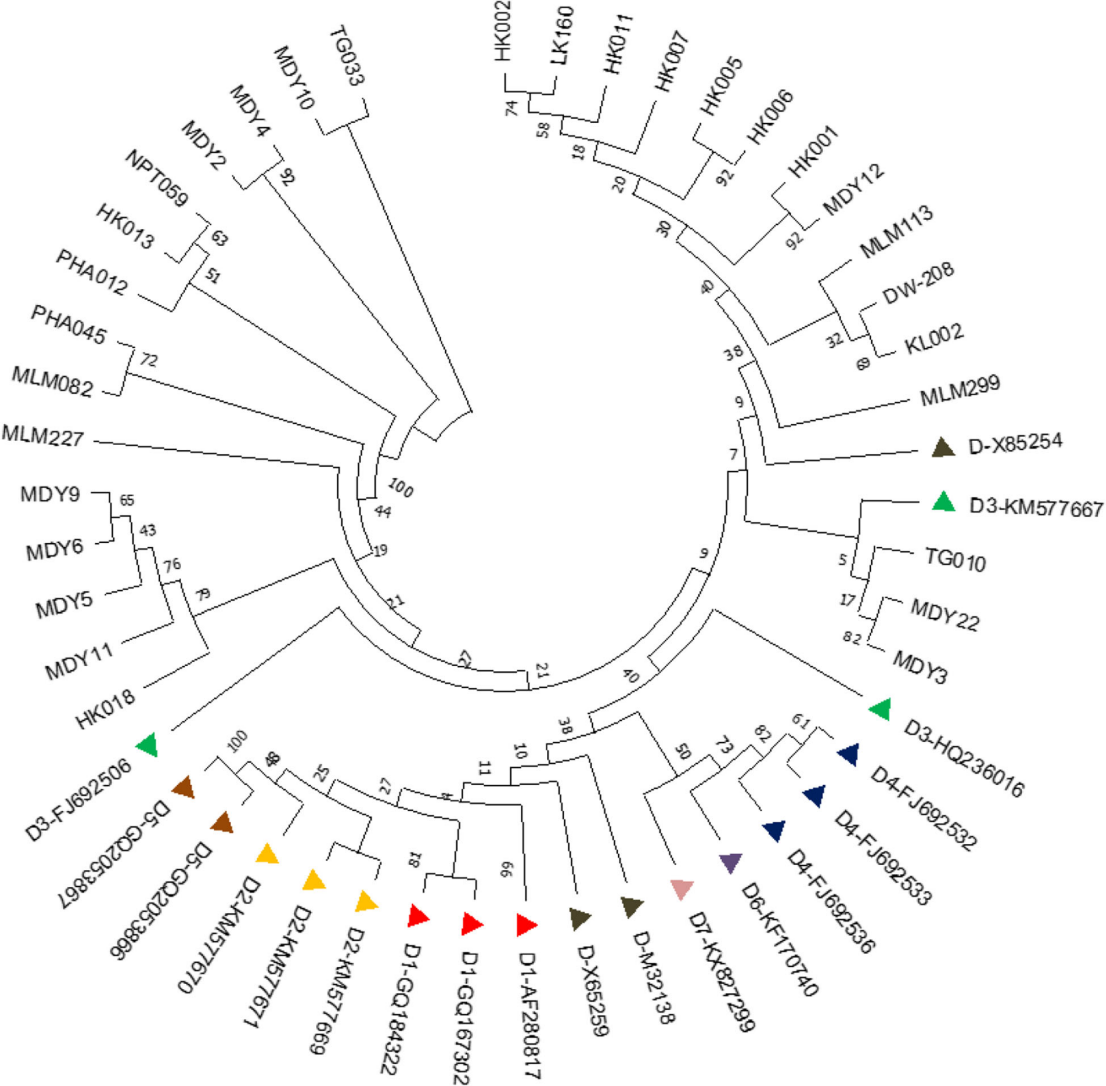
Cladogram of HBV sub-genotypes of genotype D. Phylogenetic tree was constructed using 578 bp nucleotide sequences (2860–222) PreS1/ PreS2 region in the MEGA X, using the Maximum Likelihood method with bootstrap test of 1000 replicates and Kimura two parameter model. GenBank reference sequences are shown as HBV sub-genotype and accession number. Study sequences were designed by study number

**Fig. 5:**
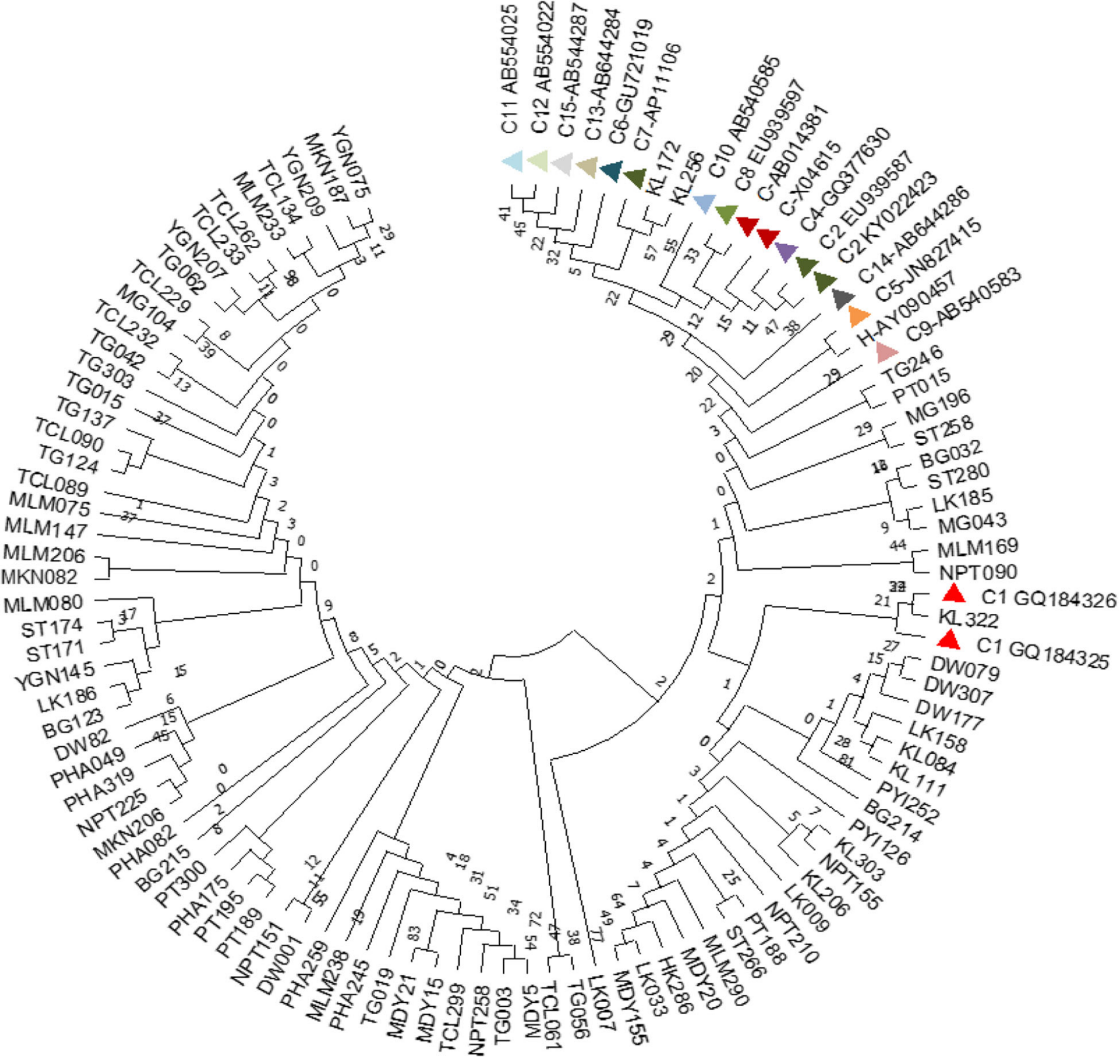
Cladogram of HBV sub-genotypes of genotypes C. Phylogenetic tree was constructed using 578 bp nucleotide sequences (2860–222) PreS1/ PreS2 region in MEGA X using the neighbor-joining method with bootstrap test of 1000 replicates and maximum composite likelihood model. GenBank reference sequences are shown as HBV sub-genotype and accession number. Study sequences were designed by study number

Genotype D study sequences were clustered into sub-genotype D3 by reference sequences of HBV sub-genotypes D1 to D8 retrieved from the GenBank data base, together with genotype D study sequences, to construct phylogenetic tree by neighbor-joining.

method using the maximum composite likelihood method to calculate evolutional distance (Fig. 4); Genotype C sequences were clustered into sub-genotypes C1, C5 and C7, as determined by the sub-genotype reference sequences (Fig. 5).

## Discussion

HBV genotyping is important to clarify the route of infection and virulence of the virus. In particular, examination of sequence diversity among different isolates of the virus is important, since variants may differ in their patterns of serological reactivity, replication of the virus, activity of liver disease, prognosis, and response to treatment. A total of 353 subjects from the general population of Myanmar having hepatitis B infection were enrolled in this study. There is no previously available information regarding the regional prevalence of HBV genotypes from Myanmar. In a multi-country study on chronic liver disease patients, the most common genotype identified in Myanmar was type C [21, 26, 27]. In the current study findings, the major genotype was HBV genotype C, which is in accordance with previous HBV studies in Myanmar. HBV genotypes are known to have a divergent geographic distribution. The predominant genotypes reported from Southeast Asian countries were genotype C from Thailand, and genotypes C and B from Indonesia [28]. In China, HBV genotype C and B were found to be predominant among the Negrito and Mongoloid tribes. Moreover, HBV genotype A and D were the most prevalent in India [14]. In this study, HBV genotype D is the predominant genotype in the western area of Myanmar, and type C was mostly found in the eastern region bordering China and Thailand, as well as in the central and southern areas, indicating that genotype C, the major genotype of Mongoloid tribes, might be the causative agent of infection.

Moreover, Paraskevis et al. [29] reported that genotype C is the oldest HBV genotype and has the highest numbers of sub-genotypes, viz., C1-C16 [30, 31], reflecting the long duration of its endemicity in humans. In this study, we determined a few sub-genotypes circulating in different parts of Myanmar, with the majority being sub-genotype C1. This was similar to the findings of HBV sub-genotypes found in United States-bound refugees from Myanmar, and adult immigrants to Australia from Myanmar [32, 33]. Considering the sub-genotypes of HBV genotype C, at least two subtypes are found in Asia: HBV genotype C1 was found only in Southeast Asia including Vietnam, Myanmar and Thailand, while HBV genotype C2 was found in east Asia including Japan, Korea and China [27]. In this study, most genotypes of C were found to belong to the sub-genotype C1, and was equally distributed throughout Myanmar. A low percentage of strains from the study subjects showed sub-genotypes C5 and C7, mainly in the central region of Myanmar. Moreover, this result was quite similar to the previous study on 15 isolates from hepatitis carriers, which showed that all HBV isolates were sub-genotypes C1 [18], and seemed to be present for quite a long time in Myanmar [26]. Presence of multiple sub-genotypes C indicate that HBV has proliferated since long in Myanmar.

According to the recent system and comparative analysis of the sub-genotype D, at least six variants (D1- D6) have been classified. Of these, the sub-genotype D3 was most frequently determined in this study, followed by D6 (Table 1). Few incidences of genotype D were reported in previous clinical case studies, and we believe this is the first report on prevalence of HBV genotype D, sub-genotype D3 in Myanmar [16]. However, the prevalence of genotype D was higher than previous findings, which might be due to frequent international travels of individuals, and also due to migration. In addition, in Myanmar, there is no large-scale study of HBV genotypes with application of sequencing for reliable data, and the outcome may also be associated with regional variation. Genotype D was mostly found in the western region of Myanmar which is quite near India and Bangladesh, where genotype D is more prevalent [34, 35].

In the current study, a relatively lower proportion (1.3%) of the study population tested positive for genotype B, sub-genotype B4 and B5. A previous study in the Yangon region had also reported the absence of B genotype in their study population [16]; however, inconsistent findings were reported in a study of Australian adult immigrants from Myanmar, where 10.5% of the study population were characterized as genotype B [33]. In our study, only 1.3% of the study population was found to have the HBV genotype B, thereby indicating that a low prevalence of this genotype was circulating in the country.

It has been reported that the geographical distribution of HBV genotypes might be related to the route of exposure to infection. For example, HBV genotype B and C were more common in highly endemic regions like Asia and Africa in which perinatal or vertical exposure is an important route of viral transmission. Other genotypes were primarily observed in regions of horizontal exposure [11–13, 36, 37]. Therefore, HBV genotype distribution can be provided as epidemiological evidence for investigating viral acquisition and the geographical scattering pattern of HBV [11–13, 36, 37]. In the current study, genotype C was predominant in most regions of Myanmar, and vertical transmission seems to be the main mode of transmission. As there is no documented study on the transmission pattern of HBV in Myanmar, further studies are required for verifying this hypothesis.

Because of frequent international travels and human migration across countries, introduction of new HBV genotypes to a community might have far reaching effects, including recombination between genotypes [36] or replacement of one genotype by another [38]. Compared to the HBV genotype B, the genotype C is associated with delayed hepatitis B e antigen (HBeAg) seroconversion [38], more-active hepatitis [39], lower response to antiviral therapy [40], more advanced liver disease, and a higher risk of hepatocellular carcinoma [41].

In addition, we found that 102/153 subjects (66.7%) of the study population are genotype C isolates. Thus, the patients infected with genotype C need to be carefully monitored to assess their future clinical outcomes. Particularly, sub-genotype C1 is documented to have an increased tendency for the development of cirrhosis and hepatocellular carcinoma (HCC), especially in patients over 50 years of age [42–44]. On the other hand, genotype B patients have higher rates of HBeAg seroconversion, and HCC has been detected in younger patients [42, 45, 46]**.**

## Conclusions

HBV genotype C, sub-genotype C1 is the most predominant variant in Myanmar and is distributed throughout the states and regions, whereas genotype HBV genotype D (sub-genotype D3 and D6) is predominantly found at the Myanmar–India border. This study provides information on the geographical distribution of viral hepatitis B genotypes in Myanmar, and can contribute towards establishing Hepatitis B control measures in Myanmar.

## Supplementary information

**Additional file 1.**

## Data Availability

The partial sequences of 153 HBV isolates have been submitted to the Gene Bank. The accession numbers of this study isolates are MH816993–995 and MH925817–925863. Original data may be obtained by email to corresponding author.

## Abbreviations

HBV: Hepatitis B virus
HBsAg: Hepatitis B surface Antigen
ICT: Immunochromatographic test
PCR: Polymerase chain reaction
NCBI: National Centre for Biotechnology Information
WHO: World Health Organization
RT: Reverse transcriptase
HBeAg: Hepatitis B envelope antigen
PSU: Primary Sampling Unit
SSU: Secondary Sampling Unit
