# Supplementary material for: Distribution of hepatitis B virus genotypes in the general population of Myanmar via nationwide study
Source: BMC Infect Dis. 2020 Jul 29;20:552. doi: 10.1186/s12879-020-05269-z (PMC7392661; doi:10.1186/s12879-020-05269-z)
Supplement: Supplementary file 1 — Additional file 1. [file 12879_2020_5269_MOESM1_ESM.docx]

The following genotype reference strains (genotype-Genbank accession number) were used in alignment:

A-AF090842,A-X02763,A-X51970, B-D00329, B-AB 073846, B-AB-AB 602818, C-AB014381, C-X04615, D-X65259, D-M32138,D-X85254,E-AB032431, E-X75657,F-AB 036910, F-AF 223965, F-X69798, G-AF160501, G-AF 405706, H-AY 090454, H-AY 090457, H-AY 090460, B1-FJ 899799, B2- AB981582, B3- GQ 924617, B4-GQ 924626, B5-GQ 924640, B6- JN 792893, B7- GQ 358137, B8-GQ 358147, B9- GQ 358419, C1-GQ 184325, C1-GQ 184326, C2-E4 939587, C2-KY 022423, C3-EU 939596,C4-GQ377630,C5-JN 827415, C5-KM 999992,C6-GU 721029, C7-AP11106, C8-AP011106,C8-EU 939597, C9-AB 540583, C10-AB 540585, C11-AB 554025, C12-AB 644280, C13-AB 644284, C14-AB 644286, C15-AB 544287, D1-AF 280817, D1- GQ 167302, D1-GQ 184322, D2-KM 577699, D2-KM-577670, D2-KM577671, D3-FJ 692506, D3- HQ236016, D3-KM 577667, D4-FJ692532, D4-FJ 692533, D4-FJ 692536, D5-GQ 2053866, D5-GQ 2053867, D6-KF 170740, D7-KX 827299.
